# HPV Infection and EGFR Activation/Alteration in HIV-Infected East African Patients with Conjunctival Carcinoma

**DOI:** 10.1371/journal.pone.0010477

**Published:** 2010-05-17

**Authors:** Jing Jie Yu, Pingfu Fu, John J. Pink, Dawn Dawson, Jay Wasman, Jackson Orem, Walter O. Mwanda, Honglan Zhu, Xiaobing Liang, Yi Guo, William P. Petros, Ronald T. Mitsuyasu, Henry Wabinga, Scot C. Remick

**Affiliations:** 1 Mary Babb Randolph Cancer Center and Molecular Medicine Core Facility, Schools of Medicine and Pharmacy, Robert C. Byrd Health Sciences Center, West Virginia University, Morgantown, West Virginia, United States of America; 2 Departments of Biostatistics and Epidemiology, Medicine, and Pathology, Center for AIDS Research, and Case Comprehensive Cancer Center, Case Western Reserve University, Cleveland, Ohio, United States of America; 3 Uganda Cancer Institute, School of Medicine, Makerere University, Kampala, Uganda; 4 Department of Pathology, Kenyatta National Hospital, University of Nairobi, Nairobi, Kenya; 5 Department of Medicine, University of California Los Angeles, Los Angeles, California, United States of America; 6 Department of Pathology, School of Medicine, Makerere University, Kampala, Uganda; University of Kansas Medical Center, United States of America

## Abstract

**Background:**

There has been substantial growth in the numbers of patients with conjunctival squamous cell carcinoma infected with HIV in East Africa. The natural history of the conjunctival squamous cell carcinoma appears to be unique in this region of the world, but the etiologic mechanism unclear and therapeutic options limited. This research was carried out to determine if conjunctival squamous cell carcinoma harbors human papillomavirus DNA and is associated with activation of the EGFR signaling pathway. Positive findings would identify etiologic causes and provide clinical guidance to improve treatment.

**Methods/Findings:**

Expression of p-MAPK/MAPK, p-Akt/Akt and p-EGFR/EGFR in cell nuclei and cytoplasm of 38 FFPE specimens were assessed by immunohistochemistry; HPV genotype was detected by qPCR assay; EGFR mutation was assessed by DNA sequencing analysis; and EGFR mRNA expression was measured using relative qPCR. Statistical analyses included two-sided Fisher exact test or chi-square test, Spearman correlation coefficient and ANOVA. HPV 18 was found in 61% of samples, with HPV 16 double-genotype in 6 patients (16%). Immunohistochemistry and qPCR data suggest that activation and expression of the EGFR signaling pathway is related to disease progression of conjunctival cancer. The associations between cytoplasmic p-MAPK, cytoplasmic p-Akt and tumor invasiveness were significant (p = 0.05 or 0.028). Nuclear p-EGFR appeared only in invasive tumors. A significant positive association between EGFR expression and disease invasiveness was observed (p = 0.01). A SNP in 10 patients and one missense mutation were found within EGFR tyrosine kinase domain. Statistical analysis indicates that patients with measurable EGFR expression more likely harbor EGFR mutations, compared to those with negative EGFR expression (35.3% vs. 0%).

**Conclusions/Significance:**

We conclude that HPV types 16/18 infection is frequent in East African patients with AIDS-associated squamous cell carcinoma of the conjunctiva. EGFR activation/alteration may contribute to and sustain the high prevalence of this cancer. Our findings hint that adoption of HPV vaccination strategies may impact the incidence of conjunctival carcinoma. Agents that target the EGFR pathway may have potential therapeutic benefit.

## Introduction

An association between human immunodeficiency virus (HIV) infection and squamous cell carcinoma of the conjunctiva was first reported in the mid-1990s. Since then there has been a substantial increase in patients with conjunctival squamous cell carcinoma infected with HIV in East Africa [1], [2]. In 1995, Ateenyi-Agaba observed that a high incidence of these tumors in Uganda appeared to be related to HIV infection [3]. Waddell and colleagues suggested that HIV infection is strongly associated with an increase in the incidence of conjunctival carcinoma in Africa and that immunosuppression from HIV facilitates activity of other infective agents that induce the carcinoma [4]. Recently, a pathophysiologic study found that HPV types 16 and 18 play a critical role in the oncogenesis of conjunctival cancers in subtropical Tanzania [5]. Thus, conjunctival squamous cell carcinoma is of growing concern in East Africa. The natural history of this disease appears to be unique in this region of the world, though the etiologic mechanism is unclear and therapeutic options remain limited.

Human papillomaviruses (HPV) are a group of host-specific DNA viruses with 15 high-risk or oncogenic subtypes which have been shown to act as carcinogens in the development of cervical, anogenital and conjunctival squamous cell cancers. Persistent HPV infections are the major cause of cervical cancer and contribute to other cancers [6], [7]. Studies indicate that viral oncoproteins encoded by HPV can disturb cellular responses to signals emanating from growth factor-linked signal transduction pathways, such as those mediated by EGFR, an important cellular survival factor [8]. Oncoprotein E5, encoded by HPV16, enhances the activation of the epidermal growth factor receptor and its downstream signal transduction pathways through the MAP kinase activity [9]–[11]. The E6 oncoprotein, encoded by HPV16 and HPV18, is known to bind the tumor suppressor gene product p53 and promotes p53 degradation [12]. The E7 oncoprotein, encoded by HPV16 and HPV18, binds to the retinoblastoma tumor suppressor gene product pRB and results in E7-induced inactivation of pRB [13]. The E5 protein cooperates with E7 to transform cells and enhances the ability of E7 to induce proliferation, and with E6 to immortalize cells [9]. Abundant preclinical and clinical data suggest that blocking the function of EGFR can enhance the efficacy of chemotherapy and radiotherapy and promote tumor regression in epithelial and squamous carcinomas [14].

We hypothesized that a proportion of squamous cell carcinoma of the conjunctiva, a unique AIDS-associated malignancy in equatorial Africa, would harbor human papillomavirus (HPV) DNA. Given it is an epithelial malignancy, the epidemiologic and demographic similarities between Kaposi's sarcoma and lymphoma seen in the background of HIV/AIDS in this region, we also suspected there would be evidence of activation of the epidermal growth factor receptor signaling cascade. Moreover, we proposed that an exploratory tissue-based study would provide evidence for the relation of HPV infection and EGFR signaling in this tumor type and serve as a guide for clinical trials investigating targeted agents or other therapeutic strategies.

Three major methodologies have been used in the past for detection of specific types of HPV DNA in specimens from cancer patients: antibodies against various HPV types for immunohistochemistry (IHC); fluorescence *in situ* hybridization (FISH); and polymerase chain reaction (PCR) in blood, biopsies and formalin-fixed paraffin-embedded (FFPE) tissue samples [15]–[18]. These methodologies provided preliminary information to open this area of research. Unfortunately, there are conflicting reports on the classification of tumor associated HPV subtypes and unclear directions for clinical trials and therapeutic strategies due to the limited sensitivity of available assays [19]. In this investigation we applied quantitative PCR (qPCR) to classify HPV genotypes.

## Materials and Methods

### Ethics Statement

Archived FFPE samples from patients with squamous cell carcinoma of the conjunctiva were obtained from the Uganda Cancer Registry in Kampala and the Pathology Department at the Kenyatta National Hospital in Nairobi, Kenya. Before the samples were shipped for analysis, they had been stripped of all patient identifiers. There is no way of linking molecular laboratory data with any subject in this study. Accordingly, ethical approval was not necessary as there were no risks to human subjects. Furthermore, no clinical data was abstracted from the medical records and at time of retrieval most of these patients were presumed dead.

### Human Samples

We examined 38 FFPE specimens obtained from Ugandan and Kenyan patients with documented HIV infection and biopsy-proven conjunctival squamous intraepithelial neoplasia (spanning the spectrum of dysplasia, *in situ* disease and invasive tumor as shown in Table 1). These de-identified tumor samples were retrieved from archives in the Departments of Pathology at the Makerere University School of Medicine and Uganda Cancer Institute; the Kampala Tumor Registry; and the Kenyatta National Hospital and the University of Nairobi College, Health Sciences in Kenya. Hematoxylin and eosin stained slides were reviewed by pathologists (DD and JW) to confirm diagnosis. For detection of HPV DNA genotypes, slides were stained using the Arcturus histogene kit and areas of squamous intraepithelial neoplasia were acquired using Laser Capture Microdissection (LCM). Additional tumor tissue slides were prepared for immunohistochemistry using standard techniques. For DNA and RNA studies of EGFR mutations and quantitation of EGFR mRNA, adjacent tumor tissues around the LCM residue were collected from the tissue block.

**Table 1:** Pathological classification of Patient Specimens Infected by HPV 16/18 Genotypes.

Patient Specimens	Dysplasia	*In situ* Disease	Invasive Tumor
(Total 38)	5	22	11
HPV 16 Infection	0	3	3
HPV 18 Infection	5	12	6

### DNA Extraction

Prior to LCM, samples were de-paraffinized with xylene and sequentially washed with a graded series of ethanol solutions (100%, 95% and 75%) stained and dehydrated using the protocol and reagents from an Arcturus histogene kit. Approximately 500 to 1,000 tumor cells were captured via LCM by a pathologist using the Arcturus Veritas system (MDS Analytical Technologies, Concord, ON). Tumor DNA extraction was performed using the Pico Pure DNA Extraction kit (ARCTURUS Bioscience, Inc., Mountain View, CA). The cells were extracted by insertion of laser-captured caps into an Eppendorf tube containing digestion buffer [50 µl buffer containing 0.04% Proteinase K, 10 mM Tris-HCL (pH 8.0), 1 mM EDTA, and 1% Tween-20]. The equilibrated cells were incubated in the above reaction for 24 hr at 65°C. After 5-min centrifugation, the DNA-containing solution was heated at 95°C for 8 min to inactivate proteinase K.

### RNA Isolation and cDNA Generation

The total RNA was extracted using the RecoverAll Total Nucleic Acid Isolation kit (Ambion Inc., Austin, TX) according to the manufacturer's protocol. In brief, a 1–1.5 mm^3^ tumor section of the sample was collected in a microtube, deparaffinized with 100% xylene at 50°C for 3 min, and washed with 100% ethanol. Then, 4 µl protease K and 200 µl digestion buffer were added to each sample and incubated at 65°C for 3 hr. Isolation additive/ethanol mixture was applied and then the mixture was passed through a filter cartridge. DNase mix was added to the buffer-washed cartridge for 30 min. The elute RNA was collected using 30 µl nuclease-free water and the RNA was stored at −80°C. cDNAs were generated from 5 µg of total RNA per sample via SuperScript II reverse transcriptase (Invitrogen Corp., Carlsbad, CA).

### Real-Time Quantitative PCR with TaqMan Chemistry for HPV genotyping

HPV 16 and HPV 18 were selected because they are the common high-risk types in several cancers [9]–[12]. HPV 59 and HPV 52 were the most and the next most frequently detected high-risk types in the external genitalia [20]. These four genotypes were determined using a TaqMan-based quantitative PCR analysis. Type-specific primers and probes for HPV types 16, 18, 52 and 59 were selected to target genome segments of the E6/E7 region as previously reported [21]. These primers and FAM-labeled probes were synthesized by Applied Biosystems (Forster City, CA). All reactions were undertaken in a final volume of 20 µl containing TaqMan® Gene Expression Master Mix, tumor genomic DNA and HPV type-specific primers and probe. Amplification was performed in separate reactions for each of the 4 HPV types in a 96-well plate. The PCR was run 2 min at 50°C, 10 min at 95°C, and 40 cycles of 15 seconds at 95°C and 1 min at 60°C on an ABI 7500 Fast Real-Time PCR System. A high PCR efficiency for each component PCR was verified by analyzing serial dilution of synthesized standard DNA containing targeted segments of each HPV type, sized 74 -110 base pairs (synthesized by Sigma Life Sciences, St. Louis, MO). Additional two-triplicates containing all PCR components except template DNA were denoted no template control (NTC) to ensure that the reagents were free of contamination. Finally, Ct (threshold cycle), ΔCt, ΔΔCt, AQ or RQ and gene expression plots were generated by the 7500 Fast System SDS Software (Version 1.4). In this study, a Ct value was <35 considered as positive expression, and vice versa.

### Absolute Quantitation (AQ) for HPV genotyping

For AQ detection in addition to the above procedures, we used Hela-cell genomic DNA for HPV type 18 and synthesized DNA for types 16, 52 and 59 for standard curves. The curve was drawn by 5 serial dilutions of the above known-input-target copies (3 replicates for each concentration) *vs.* the corresponding *C*t values using the least-squares fit method. Dissociation protocol was applied to confirm the targeted PCR products. Given the standard curve, the system software interpolates the absolute quantity of target in each sample.

### Relative Quantitation (RQ) for HPV genotyping

For RQ detections, RNase-P Master Mix was used as endogenous control in duplicate in the 20 µl reaction. The Ct and comparative ΔΔCt were automatically calculated for the expression of the gene and normalized to mean Ct value of RNase-P. The RQ value was calculated using ΔΔCt method. Fold change in gene expression was calculated as 2^− ΔΔCt^.

### EGFR mRNA Expression by Real-Time RQ PCR

cDNA generated from 5 µg of total RNA for each sample was used in this assay. Primers and FAM-labeled probes of the tyrosine kinase domain (exons 20–21) of the EGFR gene were purchased from Applied Biosystems. Human 18S rRNA was applied in duplicate as endogenous control. The remaining procedures were as described for RQ PCR for HPV detection.

### EGFR Mutation Analyses

Tumor DNA was amplified by polymerase chain reaction with primers for each exon (18, 19, 20 and 21) of the EGFR tyrosine kinase domain and AmpliTaq DNA polymerase, FS. In brief, five nanograms of DNA were used for the first PCR reaction and 2.5 µl of the first PCR products were applied for nested PCR [22]. PCR amplicons were separated on agarose gel and purified using a QIAQuick Gel Extraction Kit (Qiagen, Germantown, MD). The purified fragments were sequenced to identify EGFR mutations on a CEQ 8000 Genetic Analysis System with DTCS Quick Start kit (Beckman Coulter, Fullerton, CA). All sequence variants were confirmed in duplicate independent PCR amplifications and sequencing reactions to insure that detected mutations were not the result of PCR artifact.

### MAP Kinase, Akt and EGFR Immunohistochemistry

Immunohistochemistry for MAP Kinase, Akt and EGFR were performed on FFPE tissue. Sections (5 µm thick) on positively stained slides were deparaffinized, hydrated and pretreated for antigen retrieval in 10 mmol/l citrate buffer (pH 6.0) in a steamer at 98°C for 45 min. Staining was performed using polyclonal rabbit anti-P44/42 and anti-Phospho-P44/42 MAP Kinases or anti-Akt (Ser473) and anti-p-Akt (Ser473) antibodies (1∶100 dilution) or monoclonal rabbit anti-EGFR (1∶25 dilution) and anti-phospho-EGFR (Tyr1173; 1∶50 dilution) from Cell Signaling Technology, Inc. (Danvers, MA), DAKO EnVision systems (DAKO Corporation, Carpinteria, CA), and chromogen 3,3′-diaminobenzidine tetrahydrochloride. The staining intensity of the conjunctival squamous cell carcinoma was compared with that of adjacent non-neoplastic tissue by semiquantitative visual evaluations by two observers (DD and JW). In immunohistochemistry data from these patients, we utilized the standard convention staining of 3 and 4 scores as positive, and of 1 and 2 as negative for statistical analysis.

### Statistical Analysis

The association between two factors was examined by Fisher exact test or chi-square test. The association between two ordinal variables was estimated by Spearman correlation coefficient. The difference of the ratio of phospho-MAPK (Akt, EGFR) to total MAPK (Akt, EGFR) among 3 types of tissues (dysplasia, *in situ* disease and invasive tumor) was examined by ANOVA. All statistical analyses were two-sided and done using SAS (SAS Institute, Cary, NC), and p-value ≤0.05 was considered statistically significant.

## Results

### Prevalence of HPV infection is high in conjunctival squamous intraepithelial neoplasia of HIV-infected patients in East Africa

**Figure 1:**
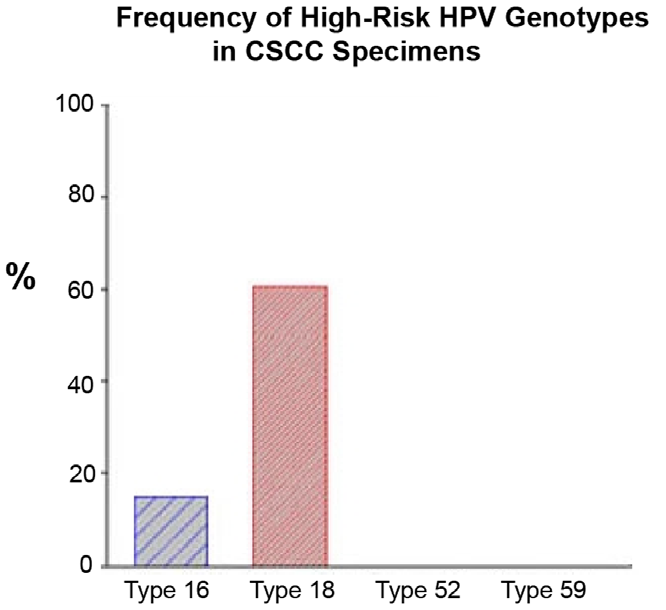
Prevalence of high-risk HPV genotypes in conjunctival squamous cell carcinoma by real-time RQ PCR assay. Tumor DNA extraction was performed using Pico Pure DNA Extraction kit. The viral DNA of 4 HPV genotypes were determined using a TaqMan-based real-time quantitative PCR analysis. Type-specific primers and probes for HPV types 16, 18, 52 and 59 were selected to target genome segments of the E6/E7 region and synthesized by Applied Biosystems. RNase-P as endogenous control for each sample (in duplicate) was applied in this assay. Amplification results from the endogenous control were used to normalize the amplification results from the target HPV types. Finally, Ct, ΔCt, ΔΔCt, RQ and gene expression plots were generated by the ABI 7500 Fast System SDS Software (Version 1.4).

Viral DNA of high-risk HPV genotypes 16 and 18 was detected in surveyed specimens by absolute- and relative-quantitation real-time PCR assays. HPV 18, the most common oncogenic strain, was found in 23 of 38 (61%) conjunctival cancer patients. Three *in situ* diseases and 3 invasive tumors of the 23 HPV 18 positive patients also showed coincident HPV 16 infection (i.e., double genotypes of HPV 16 and 18). High-risk HPV types 52 and 59 were not characterized in these samples (Table 1 & Fig. 1). This technique appears to be a sensitive, reproducible and reliable method to determine HPV genotypes in FFPE samples of conjunctival squamous cell carcinoma.

### Activation of EGFR signaling pathway is related to disease progression of conjunctival squamous cell carcinoma

Nuclear and cytoplasmic expression of phosphorylated and total EGFR and two downstream effectors of the EGFR signal-transduction pathway, MAPK and Akt, were assessed in tissue specimens by immunohistochemistry. We observed p-MAPK/MAPK and p-Akt/Akt in both the cytoplasm and nuclei of cells, in contrast to p-EGFR/EGFR, which was observed only in the nuclei. Statistical analyses of immunohistochemistry data demonstrated significant relationships between phosphorylated-MAPK or phosphorylated-Akt and disease invasiveness (Tables 2 and 3). The associations between cytoplasmic p-MAPK, cytoplasmic p-Akt and tumor invasiveness were significant (p = 0.05 or 0.028). In addition, there was some evidence that nuclear p-Akt was predictive of tumor invasiveness (p = 0.089) (Table 4). In tumors expressing nuclear p-Akt, 72.2% were invasive compared to 44.4% in tumors without nuclear p-Akt (Table 4). Though our results for EGFR protein expression did not show a direct correlation, nuclear p-EGFR staining was only present in invasive cancers (Table 5).

**Table 2:** Association between cytoplasmic phospho-MAPK and pathological classification.

Cytoplasmic p-MAPK	Dysplasia (row %)	*In situ* (row %)	Invasive cancer (row %)	p-value
IHC Score 1 and 2 (Negative)	4 (20)	8 (40)	8 (40)	**0.05**
IHC Score 3 and 4 (Positive)	0 (0)	3 (20)	12 (80)	

**Table 3:** Association between cytoplasmic phospho-Akt and pathological classification.

Cytoplasmic p-Akt	Dysplasia (row %)	*In situ* (row %)	Invasive cancer (row %)	p-value
IHC Score 1	2 (9.5)	11 (52.4)	8 (38.1)	
IHC Score 2	1 (12.5)	0 (0)	7 (87.5)	**0.028**
IHC Score 3	0 (0)	0 (0)	1 (100)	
IHC Score 4	1 (25)	0 (0)	3 (75)	

**Table 4:** Association between nuclear phospho-Akt and pathological classification.

Nuclear p-Akt	Dysplasia (row %)	*In situ* (row %)	Invasive cancer (row %)	p-value
IHC Score 1 and 2 (Negative)	3 (16.7)	7 (38.9)	8 (44.4)	0.089
IHC Score 3 and 4 (Positive)	1 (5.6)	4 (22.2)	13 (72.2)	

**Table 5:** Association between nuclear phospho-EGFR and pathological classification.

Nuclear p-EGFR	Dysplasia (row %)	*In situ* (row %)	Invasive cancer (row %)	p-value
IHC Score 1	0 (0)	0 (0)	9 (100)	
IHC Score 2	0 (0)	0 (0)	6 (100)	NA
IHC Score 3	0 (0)	0 (0)	6 (100)	

### EGFR mRNA expression is significantly correlated with tumor invasiveness in conjunctival squamous cell carcinoma patients

We investigated EGFR mRNA expression in these samples by real-time RQ PCR using primers and probes selected within the tyrosine kinase domain (exons 20–21) of the EGFR gene. In this series, EGFR mRNA expression was measured in tissues from 22 patients. Seventeen of 22 patients (77%) showed EGFR mRNA expression (Table 6). We also observed that EGFR mRNA expression correlated with nuclear p-EGFR protein expression (data not shown). As expected, a significant positive association between EGFR expression and disease invasiveness was observed (p = 0.01). Of all EGFR positive tumors 70.6% were invasive, while 0% of the EGFR negative tumors were invasive (Table 6).

**Table 6:** Association between EGFR mRNA expression and pathological classification.

EGFR mRNA expression (Total 22)	Dysplasia (row %)	*In situ* (row %)	Invasive cancer (row %)	p-value
Not Expressed (−)	1 (20)	4 (80)	0 (0)	**0.01**
Expressed (+)	1 (5.9)	4 (23.5)	12 (70.6)	

### Patients with EGFR expression are more likely to have EGFR alteration

EGFR mutational status was assessed in the patient tumor DNA of the conjunctival squamous cell carcinoma through DNA sequencing analysis. Among the 38 assessed samples, one missense mutation was found; a C to T transversion within exon 20 of the EGFR gene, resulting in a substitution of the 767 alanine for a valine. In addition, a G to A transition within EGFR exon 20 was detected in 10 of the 38 examined samples, resulting in a SNP (Silent Gln 787 Gln; data not shown). No other sequence alterations were detected in exons 18, 19 and 21 of the EGFR tyrosine kinase domain in these samples. Our analysis indicated no statistically significant association between EGFR expression and EGFR exon-20 mutation (Table 7). However, samples with positive EGFR expression were more likely to have EGFR mutation, compared to those with negative EGFR expression (35.3% vs. 0%) (Table 7).

**Table 7:** Patients with EGFR expression are more likely to have EGFR mutation.

	EGFR Exon-20 mutation		
EGFR expression	Wild Type (row %)	Mutation (row %)	p-value
Not Expressed (−)	5 (100)	0 (0)	0.266
Expressed (+)	11 (64.7)	6 (35.3)	

## Discussion

The EGFR gene is located on chromosome 7p12 and codes for a 170 kD receptor, present on the membrane of cells as inactive monomers. Upon ligand binding to the extracellular domain, the receptor undergoes conformational changes, dimerizes and becomes autophosphorylated in key tyrosine residues in the intracellular tyrosine kinase domain. This leads to the activation of downstream pathways, including MAPK and PI3K/Akt pathways, which control cell survival, inhibition of apoptosis, and proliferation [23]. EGFR-mutation is one of the most frequent genetic changes found in human cancers [24]. Much attention has been given to the oncogenic effect of the EGFR gene and EGFR-targeted therapies.

This study used phospho-specific antibodies directed at activated EGFR and downstream effectors to characterize expression, state of constitutive activation and subcellular translocation. The results revealed the constitutive activation and overexpression of the MAPK/Akt pathways. Specifically, we observed 1) increased expression of phosphorylated (Tyr1173), nuclear EGFR; the upstream tyrosine kinase that transduces signals through both the PI3K/Akt and MAPK pathways, thereby contributing to constitutive activation of these signaling pathways in conjunctival squamous cell carcinomas and 2) correlative downstream overexpression of nuclear -phospho-P44/42 MAPK and phospho-Akt (Ser473). These observations are consistent with the findings of Shepler's group that there is frequent EGFR amplification and MAPK and Akt phosphorylation in conjunctival squamous cell carcinomas; of Chen and colleagues and Kohrenhagen's group that increased expression of p-MAPK and p-Akt appeared during tumor progression of cervical neoplasms [25]–[27].

Development of adenocarcinoma of the uterine cervix and squamous cell carcinoma is strongly linked to infection by high-risk HPV types [28], [29]. There is molecular evidence that viral oncoproteins (E5, E6 and E7) found in high-risk HPV genotypes inactivate the tumor suppressor proteins p53 and pRb, promote genomic rearrangement, and confer replicative and immortalizing activities in cervical neoplasms, conjunctival squamous cell carcinoma and other cancers [12], [13], [30], [31].

Our studies indicate a possible mechanism by which the oncoproteins E5, E6 and E7, encoded by high-risk HPV genotypes, activate EGFR signal transduction and lead to increased signaling through the MAPK/Akt pathways, resulting in unchecked cellular proliferation. In other words, activation of the EGFR pathway in conjunctival squamous cell carcinoma appears to play a role in genetic alteration to maintain a malignant phenotype, as a consequence of the genomic instability associated with HPV infection. These findings are consistent with our understanding of the effects of HPV, the principle etiologic agent associated with EGFR activity, in providing etiopathogenic insight into HIV-infected conjunctival carcinoma development.

In conclusion, our study demonstrated that TaqMan real-time qPCR assay is a sensitive and reliable method for detection of specific HPV genotypes in formalin-fixed, paraffin-embedded tumor samples. Our findings also suggest that constitutively activated and overexpressed MAPK/Akt, acting in concert with EGFR overexpression/alteration, and in collaboration with viral oncoproteins, play a key role in conjunctival carcinoma oncogenesis. Further investigation of the mechanisms of HPV-oncoprotein effects on EGFR activation/alteration is warranted. Our findings hint that adoption of HPV vaccination strategies may impact the incidence of conjunctival squamous cell carcinoma and use of agents that target the epidermal growth receptor tyrosine kinase pathway may have potential therapeutic benefit.
